# The Quantitative Assessment of Using Multiparametric MRI for Prediction of Extraprostatic Extension in Patients Undergoing Radical Prostatectomy: A *Systematic Review and Meta-Analysis*

**DOI:** 10.3389/fonc.2021.771864

**Published:** 2021-11-22

**Authors:** Wei Li, Yuan Sun, Yiman Wu, Feng Lu, Hongtao Xu

**Affiliations:** ^1^ Department of Medical Imaging, Jiangsu Vocational College of Medicine, Yancheng, China; ^2^ Department of Burn and Plastic Surgery, 71st Group Army Hospital of People’s Liberation Army of China, Xuzhou, China; ^3^ Department of Radiology, Wuxi No. 2 People’s Hospital, Wuxi, China

**Keywords:** magnetic resonance imaging, prostate neoplasms, quantitative, meta-analysis, tumor size

## Abstract

**Purpose:**

To investigate the diagnostic performance of using quantitative assessment with multiparametric MRI (mpMRI) for prediction of extraprostatic extension (EPE) in patients with prostate cancer (PCa).

**Methods:**

We performed a computerized search of MEDLINE, Embase, Cochrane Library, Web of Science, and Google Scholar from inception until July 31, 2021. Summary estimates of sensitivity and specificity were pooled with the bivariate model, and quality assessment of included studies was performed with the Quality Assessment of Diagnostic Accuracy Studies-2. We plotted forest plots to graphically present the results. Multiple subgroup analyses and meta-regression were performed to explore the variate clinical settings and heterogeneity.

**Results:**

A total of 23 studies with 3,931 participants were included. The pooled sensitivity and specificity for length of capsular contact (LCC) were 0.79 (95% CI 0.75–0.83) and 0.77 (95% CI 0.73–0.80), for apparent diffusion coefficient (ADC) were 0.71 (95% CI 0.50–0.86) and 0.71 (95% CI 059–0.81), for tumor size were 0.62 (95% CI 0.57–0.67) and 0.75 (95% CI 0.67–0.82), and for tumor volume were 0.77 (95% CI 0.68–0.84) and 0.72 (95% CI 0.56–0.83), respectively. Substantial heterogeneity was presented among included studies, and meta-regression showed that publication year (≤2017 *vs.* >2017) was the significant factor in studies using LCC as the quantitative assessment (P=0.02).

**Conclusion:**

Four quantitative assessments of LCC, ADC, tumor size, and tumor volume showed moderate to high diagnostic performance of predicting EPE. However, the optimal cutoff threshold varied widely among studies and needs further investigation to establish.

## Introduction

Extraprostatic extension (T3a and T3b) in PCa is associated with a higher risk of biochemical recurrence and metastatic disease after radical prostatectomy (RP) or radiotherapy (1, 2). Although patients who undergo RP have shown high cancer-specific survival, they have a risk of suffering from postoperative erectile dysfunction and urinary incontinence (3). Preservation of the neurovascular bundles (NVB) can improve postoperative potency rate, however, which may increase the risk of positive surgical margins, bringing about biochemical recurrence and treatment failure (4, 5). Therefore, comprehensive risk assessment and staging is of great importance, which will influence the treatment planning and management. To overcome this problem, various nomograms and guidelines were proposed to improve the preoperative risk evaluation, including Partin tables, Memorial Sloan Kettering Cancer Center nomograms, and the cancer of the prostate risk assessment score (6–8). However, these well-established measures are roughly correlated with the final pathologic stage and lacking accuracy in clinical practice (9, 10).

In recent years, mpMRI has been widely applied in detection, staging, and localization of prostate cancer (PCa). In 2012, the European Society of Urogenital Radiology (ESUR) introduced Prostate Imaging Reporting and Data System (PI-RADS) for performing, interpreting, and reporting the PCa with mpMRI (11–13), which was validated and widely used in clinical practice (14, 15). Nevertheless, for localized advantage PCa of EPE, the ESUR PI-RADS demonstrated moderate diagnostic accuracy, and mainly depended on radiologists’ own experience then short of reproducibility and inter-reader agreement (16, 17). At present, quantitative assessments of EPE with mpMRI have been intensively studied and demonstrated the potential of improving accuracy, inter-reader agreement, and pathology correlation (18, 19). In PI-RADS version 2.1, quantitative metrics such as length of capsular contact (LCC), apparent diffusion coefficient (ADC), tumor size, and tumor volume were included for assisting in prediction of EPE (13). However, these parameters have not been evaluated systematically up to date. Thus, the purpose of our study was to assess the diagnostic accuracy of using quantitative metrics for the prediction of EPE.

## Methods and Materials

This systematic review and meta-analysis was performed in accordance with the preferred reporting items for systematic reviews and meta-analyses (PRISMA) guidelines (20). The primary outcome was the diagnostic performance of using mpMRI quantitative metrics of LCC, ADC, tumor size, and tumor volume as independent predictors for prediction of EPE in PCa.

### Search Strategy and Selection Criteria

For this systematic review, we carried out an electronic database search of MEDLINE, Embase, Cochrane Library, Web of Science, and Google Scholar from inception until July 31, 2021, with language restricted to English. The searches were supplemented by screening references from the most recent reviews and eligible studies. The search terms combined acronyms used for MRI, PCa, EPE, and quantitative assessments as follows: ([MR] or [MRI] or [mpMRI] or [magnetic resonance] or [magnetic resonance imaging]) and ([prostate cancer] or [PCa] or [prostate carcinoma]) and ([EPE] or [extraprostatic extension] or [ECE] or [extracapsular extension]) and ([tumor size] or [tumor volume] or [tumor dimension] or [ADC] or [apparent diffusion coefficient] or [LCC] or [TCL] or [length of tumor capsular contact] or [capsule contact length] or [tumor contact length]).

### Inclusion Criteria

We included studies that met all criteria as follows: (1) patients underwent mpMRI for assessment of suspected EPE; (2) with quantitative metric of LCC, ADC, tumor size, and tumor volume as independent predictors; (3) reported the true positive (TP), false positive (FP), false negative (FN), and true negative (TN), or other details for the reconstruction of 2×2 tables to evaluate the diagnostic performance; and (4) with pathological results after radical prostatectomy as the reference standard.

### Exclusion Criteria

We excluded studies that satisfied any of the following criteria: (1) studies involving less than 10 participants, (2) did not use the quantitative metrics as an independent predictor but combined with other scoring system or guidelines, (3) not reported sufficient for assessing the diagnostic performance, and (4) review articles, guidelines, consensus statements, letters, editorials, and conference abstracts. The literature selection was performed by two investigators (LW and SY, with 8 and 11 years of experience in performing systematic reviews and meta-analyses) independently. All disagreements were resolved by discussion and consultation with a third investigator (WM) until consensus was reached.

### Data Extraction and Quality Assessment

We used a standardized form to extract information from individual studies as follows: (1) demographic and clinical characteristics, including sample size, patient age, PSA level, and Gleason score, number of patients diagnosed with EPE; (2) study characteristics, including authors, year of publication, affiliation, country of origin, duration of patient recruitment, study design, quantitative metrics used and corresponding cutoff thresholds, number of readers and their experience, blinding; and (3) technical characteristics of mpMRI, including magnetic field strength, ***b*** values, and coil type. We used the Quality Assessment of Diagnostic Accuracy Studies-2 to evaluate the quality of studies and likelihood of bias (21), in which four domains were scored for individual study: patient selection, method of the index test (parameter measurement and use of appropriate threshold to classify lesions), using pathological results as a reference standard, and flow and timing. Data extraction was performed by one investigator (LW) and confirmed by a second investigator (SY).

### Data Synthesis and Analysis

The degree of heterogeneity between studies was measured using the inconsistency index (***I*** ^2^): 0–40%, might or have no heterogeneity; 30–60%, moderate heterogeneity; 50–90%, substantial heterogeneity; and 75–100%, considerable heterogeneity (22). The summary estimates of sensitivity and specificity were calculated with the bivariate model and hierarchical summary receiver operating characteristic (HSROC) model (23, 24). The forest plots were used to graphically present the 95% confidence interval (95% CI) of sensitivity and specificity for each study. In addition, an HSROC curve with a 95% confidence region and prediction region was constructed to demonstrate the results. The Deeks’ funnel plot was used to estimate the publication bias, and statistical significance was determined by the Deeks’ asymmetry test (25).

In the light of varied cutoff values reported across included studies, multiple subgroup analyses were performed to assess the following various clinical settings: (1) use of tumor size ≥15 mm as the cutoff threshold, (2) use of the value of ADC mean, (3) use of LCC ≤10 mm as the cutoff threshold, (4) use of LCC ≤12 mm as the cutoff threshold, (5) use of LCC >10 mm as the cutoff threshold, (6) use of LCC >12 mm as the cutoff threshold. We performed meta-regression to explore the sources of heterogeneity. For studies using LCC as the quantitative metric, the following covariates were added to the bivariate model: (1) study design (prospective *vs.* retrospective), (2) patient number (≤150 *vs.* >150), (3) magnetic field strength (1.5 T *vs.* 3.0 T), (4) malignant rate (≤30 *vs.* >30%), (5) LCC length (≤10 *vs.* >10 mm, and ≤12 *vs.* >12 mm), (6) reader number (<2 *vs.* ≥2), (7) blinded to the final results (blinded *vs.* aware partial patient information), (8) publication year (≤2017 *vs.* >2017), and (9) length of tumor size (15 *vs.* >15 mm). All analyses were conducted using STATA 16.0, and statistical significance was set at ***P*** values less than 0.05.

## Results

### Literature Search

**Figure 1:**
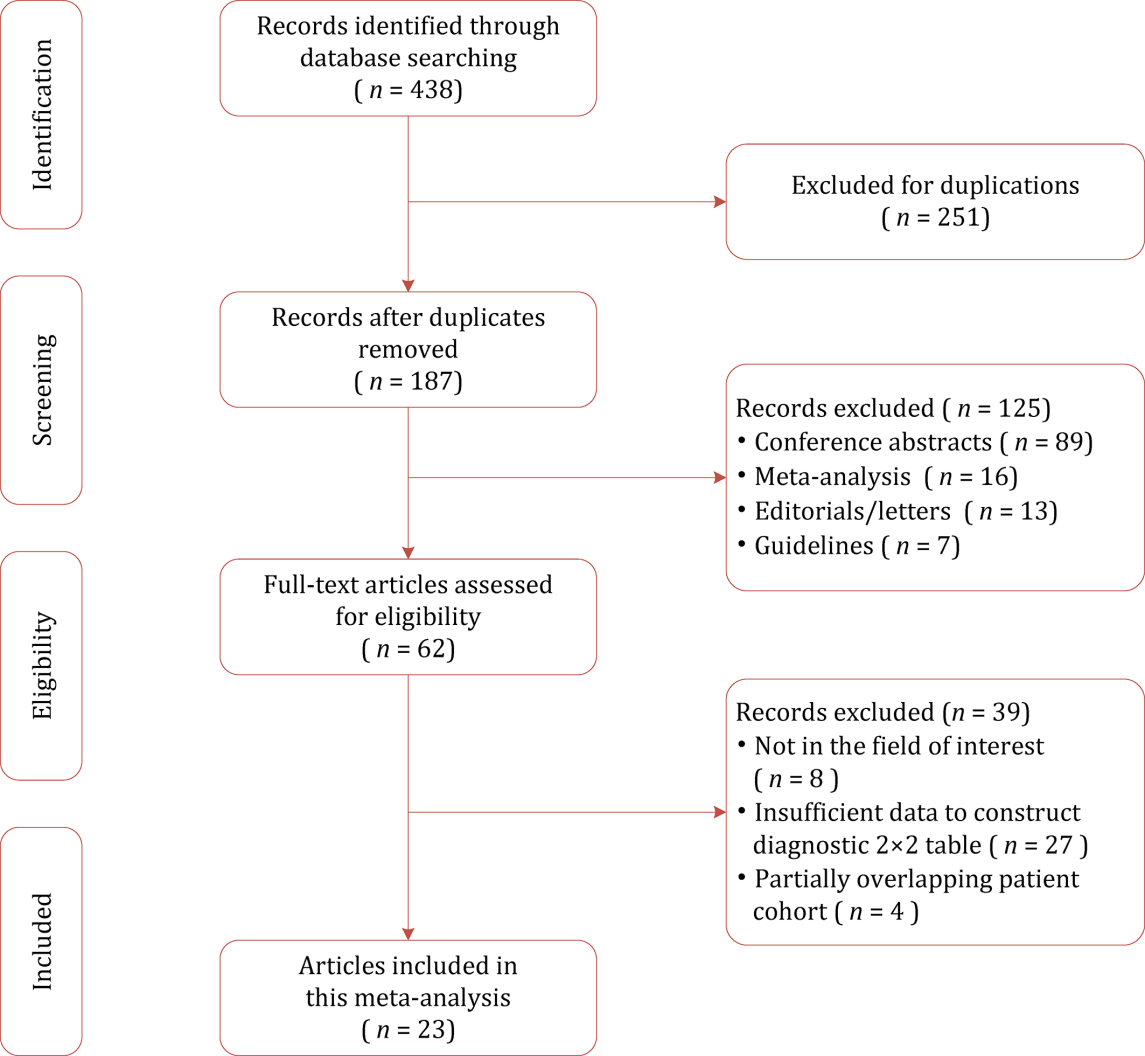
Study selection process for this systematic review and meta-analysis.

A flow chart summarizing the publication selection process is presented in **Figure 1**. Our literature search initially yielded 438 results, of which 251 were excluded owing to duplicates. After screening of titles and abstracts, a total of 125 results were excluded. Full-text analysis was performed among the remaining 62 potentially eligible articles, and 39 were excluded for reasons as follows: with insufficient data to reconstruct 2×2 tables (*n*=27), not in the field of interest (*n*=8), and partially overlapping patient cohort (*n*=4). Finally, a total of 23 studies with 3,931 participants assessing diagnostic performance of mpMRI quantitative metrics for detection of EPE were included in this study (26–48).

### Characteristics of the Included Studies

The detailed demographic characteristics are summarized in **Table 1**. The sample size of the study population ranged from 25 to 553 patients, with a mean age of 60–68 years. Based on pathological results after RP, EPE was found in 23–67% percent of participants. The PSA levels of participants ranged from 2.1 to 58.7, with a Gleason score of 5–10. In 16 studies, LCC was used for independent predictor of EPE, with cutoff values ranging from 6 to 20 mm (27–29, 31, 32, 34–36, 38, 39, 43, 44, 46–48). In three studies, tumor size was used for independent predictor, with a cutoff value of 0.9–2.1 (33, 39, 42). The diagnostic accuracy of using ADC value as independent predictor was reported by seven studies (32, 33, 35, 39–41, 45). In five studies, tumor volume was used as independent predictor, with cutoff thresholds ranging from 15 to 19 mm (26, 27, 35, 37, 46). Regarding study design, only four studies (34, 36, 38, 39) were prospective, and all of the remaining 19 studies were retrospective in nature. In 18 studies, the MRI was performed with 3.0 T scanners, whereas in the remaining five studies, MRI was performed with 1.5 T scanners (28, 32, 33, 36, 39). The MRI images were interpreted by one to three radiologists, with experience of 2–23 years. Most studies reported that radiologists were blinded to final pathological results; however, in seven studies, the readers were aware that patients had PCa (30–32, 40, 41, 45, 46). The study characteristics are summarized in **Table 2**.

**Table 1:** Demographic characteristics of the included studies.

First Author	Country	Year	Period	Patients number	Malignant	Age (year, mean ± SD/median, range)	PSA (ng/ml, mean ± SD)	GS(Range)
**Abreu-Gomez et al.** (26)	Canada	2020	2012/2018	267	223	63 ± 6	11.7 ± 10.8	≥6
**Ahn et al.** (27)	Korea	2019	Jan. 2011/Dec. 2016	221	69	<75	16.7 ± 17.4	6–9
**Baco et al.** (28)	Norway	2014	Jan. 2010/Jul. 2013	111	40	64 (45–75)	8.9 (2.5–44.0)	6–9
**Bakir et al.** (30)	Turkey	2020	2012/2018	86	24	62.5 ± 6.2	7.52 (2.1–40.0)	NA
**Caglic et al.** (48)	UK	2019	Sep. 2014/Jan. 2017	75	48	64.5 (57.2–67)	8.5 (5.7–10.4)	6–9
**Costa et al.** (29)	USA	2018	Nov. 2015/Jul. 2016	80	46	64 ± 8	8.0 ± 6.1	≥6
**Dominguez et al.** (32)	Colombia	2018	May. 2011/Dec. 2013	79	33	61.1 ± 7.5	7.0 ± 7.25	6–9
**Kim et al.** (40)	Korea	2014	Feb. 2006/Apr. 2008	167	23	66.5 (52–78) ^*^	8.5 (1.1–37.3) ^*^	6–9
**Kim et al.** (41)	Korea	2016	Dec. 2013/Jan. 2015	292	111	64.5 (42–79) ^*^	10.4 (0.13–58.7) ^*^	6–10
**Krishna et al.** (35)	Canada	2017	Nov. 2012/May. 2015	149	92	62.8 ± 6.1	7.8 ± 7.0	6–9
**Lim et al.** (42)	Canada	2015	Jan. 2012/Jun. 2014	73	38	62.8 ± 5.7	10.7 ± 10.6	6–9
**Lim et al.** (43)	Canada	2016	May. 2012/May. 2015	113	76	63 ± 5.8	8.8 ± 9.3	6–9
**Matsuoka et al.** (36)	Japan	2017	Aug. 2007/Mar. 2015	210	56	67 (50–81)	7.0 (2.9–30.0)	5–10
**Mehralivand et al.** (38)	USA	2019	Jun. 2007/Mar. 2017	553	125	60 ± 8	6.3 (0.2–170)	6–10
**Rud et al.** (39)	Norway	2018	Dec. 2009/Jun. 2012	183	103	65 (60–68)	7.9 (5.8–11.5)	6–9
**Kongnyuy et al.** (34)	USA	2016	May. 2017/Dec. 2015	397	87	60.0 (38–76)	5.5 (0.1–55.7)	≥6
**Park et al.** (46)	Korea	2020	Jul. 2016/Mar. 2017	301	129	65 ± 7	7.6 ± 5.6	6–10
**Woo et al.** (45)	Korea	2015	Jan. 2013/Dec. 2013	117	50	68.0 ± 6.8	12.2 ± 13.1	6–10
**Woo et al.** (44)	Korea	2016	Jan. 2012/Dec. 2012	185	51	66.7 ± 7.0	10.2 ± 13.6	6–9
**Schieda et al.** (37)	Canada	2016	Jan. 2012/Dec. 2015	25	13	65.0 ± 5.9	9.9 ± 7.7	6–9
**Giganti et al.** (33)	Italy	2016	NA	70	23	64 (58.9–70.5)	6.8 (5.0–9.9)	6–10
**Onay et al.** (31)	Turkey	2019	2012/2017	105	24	62 (40–77) ^*^	7.95 (2.1–46.0) ^*^	NA
**Rosenkrantz et al.** (47)	USA	2015	NA	90	23	64 ± 8	9.0 ± 11.4	6–9

NA, not available; SD, standard deviation; PSA, prostate serum antigen; GS, Gleason score.

^*^mean, range.

**Table 2:** Study Characteristics of Included Studies.

First Author	Design	Readers	Experience(Year)	Magnet field strength	*b* Values (mm/s^2^)	Coil	Blinded	Assessment metric	Cutoff threshold^†^
**Abreu-Gomezet al.** (26)	Retrospective	2	13/17	3.0 T	0/500/1,000	Surface	Yes	Size	15/19
**Ahnet al.** (27)	Retrospective	2	23/19	3.0 T	0/100/1,000	Cardiac	Yes	LLC/Size	10–16/18
**Baco et al.** (28)	Retrospective	1	>700 cases	1.5 T	0/500/2,000	Surface	NA	LLC	20
**Bakir et al.** (30)	Retrospective	3	3/6	3.0 T	0–800	Surface	Yes^*^	LLC	15.2/16.1
**Caglic et al.** (48)	Retrospective	1	8	3.0 T	150/750/1,400/2,000	PAC	Yes	LLC	10.5/13.5
**Costa et al.** (29)	Retrospective	3	NA	3.0 T	0–2,000	ERC	Yes	LLC	10
**Dominguez et al.** (32)	Retrospective	2	8/14	1.5 T	NA	None	Yes^*^	LLC/ADC	12/0.87
**Kim et al.** (40)	Retrospective	2	7/9	3.0 T	1,000	PAC	Yes^*^	ADC	1.09
**Kim et al.** (41)	Retrospective	2	2/9	3.0 T	NA	PAC	Yes^*^	ADC	0.785
**Krishna et al.** (35)	Retrospective	2	11/15	3.0 T	0/500/1,000	Surface	Yes	ADC/LLC/Size	6.991/11/15
**Lim et al.** (42)	Retrospective	2	9/14	3.0 T	0/500/1,000	Surface	Yes	Volume	2.1
**Lim et al.** (43)	Retrospective	2	11/15	3.0 T	0/500/1,000	Surface	Yes	LLC	15
**Matsuoka et al.** (36)	Prospective	2	5/10	1.5 T	0/1,000/2,000	Surface	Yes	LLC	10
**Mehralivand et al.** (38)	Prospective	2	9/15	3.0 T	1,500/2,000	Cardiac	Yes	LLC	15
**Rud et al.** (39)	Prospective	1	2	1.5 T	50–1,000	Surface	Yes	LLC/Volume/ADC	13/0.9/0.89
**Kongnyuy et al.** (34)	Prospective	2	8/16	3.0 T	NA	Surface	Yes	LLC	12.5
**Park et al.** (46)	Retrospective	2	3/15	3.0 T	0/50/500/1,000	Surface	Yes^*^	LLC/Size	10/15
**Woo et al.** (45)	Retrospective	2	21/9	3.0 T	0/1,000	None	Yes^*^	ADC	0.893
**Woo et al.** (44)	Retrospective	1	22	3.0 T	0/1,000	NA	Yes	LLC	12/13/14
**Schieda et al.** (37)	Retrospective	2	11/16	3.0 T	0/500/1,000/1,500	PAC	Yes	Size	16
**Giganti et al.** (33)	Retrospective	3	NA	1.5 T	0/800/1,600	ERC	Yes	ADC/Volume	0.84/0.88
**Onay et al.** (31)	Retrospective	2	5/12	3.0 T	0–800	Surface	Yes^*^	LLC	13/13.5
**Rosenkrantz et al.** (47)	Retrospective	2	1/4	3.0 T	50 and 1,000	PAC	Yes	LLC	6

ADC, apparent diffusion coefficient; ERC, endorectal coil; LCC, length of capsular contact; NA, not available; PAC, phase-array coil; RP, radical prostatectomy; SD, standard deviation.

^*^Aware that all patients had prostate cancer.

^†^For length of capsular contact and tumor size, mm; for tumor volume, ml.

### Quality Assessment

**Figure 2:**
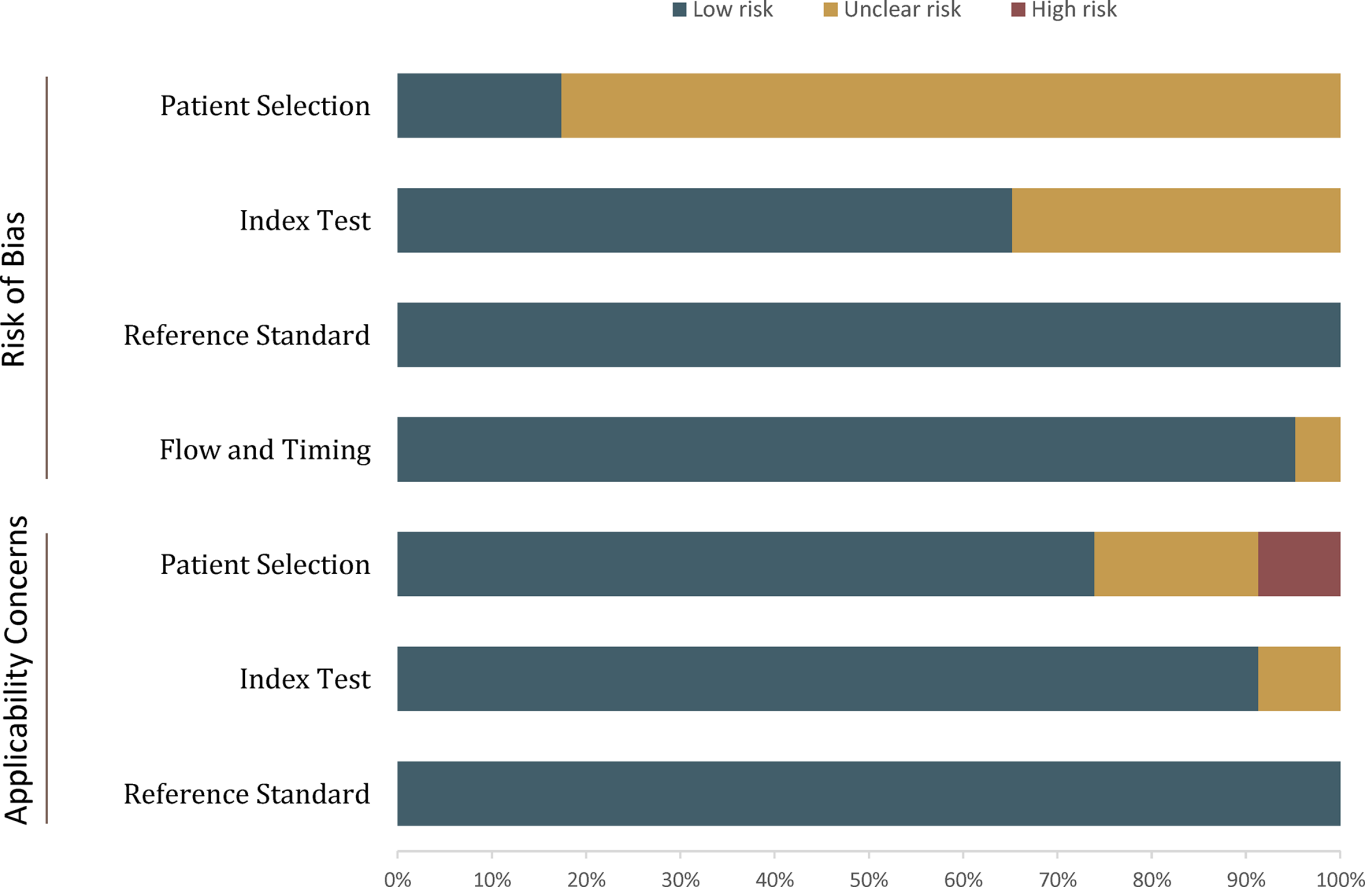
Grouped bar charts show the risk of bias and concerns for applicability of included studies.

The overall quality of the included studies was not substantially high. Concerning the patient selection domain, there was generally high risk of bias because the majority of included studies were retrospective in design (34, 36, 38, 39). In four studies, patients who classified as PI-RADS score 1–3 were excluded (26, 27, 43, 48), and in two studies, the location was restricted to the anterior prostate cancer (27, 37). Regarding the index test domain, in seven studies the radiologists were aware that patients had biopsy-proven PCa but did not know the final pathological results (30–32, 40, 41, 45, 46). One study had a concern of applicability because the blinding was not reported explicitly (28). Concerning the flow and timing domain, all studies were scored as low risk of bias as patients received the same reference standard. **Figure 2** shows the detailed quality assessment of included studies.

### Diagnostic Performance of Different Quantitative Methods

**Figure 3:**
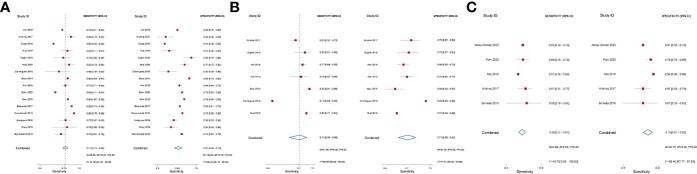
Coupled forest plot of pooled sensitivity and specificity. Numbers are pooled estimates with 95% CI in parentheses. Corresponding heterogeneity statistics are provided at bottom right corners. Horizontal lines indicate 95% confidence intervals. **(A)** Length of capsular contact; **(B)** apparent diffusion coefficient; **(C)** tumor size.

**Figure 4:**
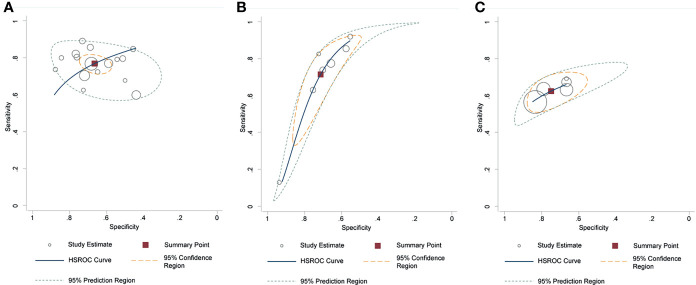
Hierarchic summary receiver operating characteristic plots with summary point and 95% confidence area for the overall. **(A)** length of capsular contact; **(B)** apparent diffusion coefficient; **(C)** tumor size.

**Figure 5:**
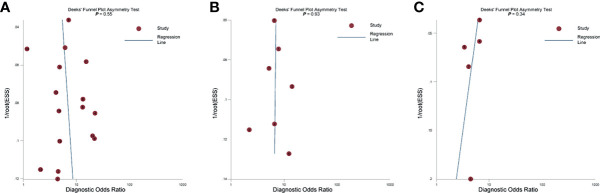
The Deeks’ funnel plot. **(A)** Length of capsular contact; **(B)** apparent diffusion coefficient; **(C)** tumor size.

The pooled diagnostic performance of LCC, ADC, and tumor size for detection of EPE is demonstrated in **Figure 3**, and the HSROC curve is presented in **Figure 4**. Regarding LCC, the pooled sensitivity and specificity were 0.79 (95% CI 0.75–0.83, ***I*** ^2^ = 67.8%) and 0.77 (95% CI 0.73–0.80, ***I*** ^2^ = 89.3%), with area under HSROC curve of 0.67 (95% CI 0.60–0.73). For ADC, the pooled sensitivity and specificity were 0.71 (95% CI 0.50–0.86, ***I*** ^2^ = 92.7%) and 0.71 (95% CI 059–0.81, ***I*** ^2^ = 77.2%), with area under HSROC curve of 0.77 (95% CI 0.73–0.80). Regarding tumor size, the pooled sensitivity and specificity were 0.62 (95% CI 0.57–0.67, ***I*** ^2^ = 19.8%) and 0.75 (95% CI 0.67–0.82, ***I*** ^2^ = 82.4%), with area under HSROC curve of 0.70 (95% CI 0.66–0.74). As for tumor volume, the pooled sensitivity and specificity were 0.77 (95% CI 0.68–0.84) and 0.72 (95% CI 0.56–0.83), with area under HSROC curve of 0.78 (95% CI 0.73–0.97). The Deeks’ funnel plot and asymmetry test demonstrated that there was no significant probability of publication bias regarding the four quantitative metrics, with ***P*** values ranging from 0.34 to 0.93 (**Figure 5**).

We performed direct comparisons between different quantitative metrics in studies providing head-to-head comparisons. Concerning LCC *vs.* ADC, the pooled summary estimates based on three studies revealed that LCC yielded significantly higher specificity as compared to ADC (0.49 *vs.* 0.79, P=0.047); however, there was no significant difference in sensitivity (0.79 *vs.* 0.55, P=0.22) (32, 35, 39). As for LCC *vs.* tumor size, the pooled summary estimates based on three studies indicated that LCC yielded significantly higher sensitivity as compared to tumor size (0.80 *vs.* 0.60, P=0.003), but at the cost of decreased specificity (0.65 *vs.* 0.78, P=0.13) (27, 35, 46). In indirect comparisons, we noted that the pooled sensitivity of LCC and tumor volume was significantly higher than tumor size, with P values of 0.002 and 0.013, respectively. Additionally, the pooled specificity for tumor volume was significantly higher than tumor size (P=0.04). Otherwise, the indirect comparisons did not identify any statistically significant differences between these four quantitative metrics (**Supplementary Table 1**).

### Subgroup Analysis and Meta-Regression

In view of different cutoff thresholds were used, we performed multiple subgroup analyses to evaluate various clinical settings. Regarding the tumor size, the pooled sensitivity and specificity were 0.72 (95% CI 0.47–0.89) and 0.70 (95% CI 0.56–0.82) for four studies using 15 mm as the cutoff threshold (26, 27, 35, 46). Regarding the ADC, the pooled sensitivity and specificity were 0.67 (95% CI 0.62–0.72) and 0.70 (95% CI 0.63–0.76) for six studies using ADC mean value (32, 33, 39–41). Regarding the LCC, the pooled sensitivity and specificity for six studies using a cutoff threshold ≤10 mm were 0.78 (95% CI 0.71–0.84) and 0.67 (95% CI 0.59–0.75), for 11 studies using a cutoff threshold ≤12 mm were 0.78 (95% CI 0.73–0.83) and 0.67 (95% CI 0.60–0.74), for 20 studies using a cutoff threshold >10 mm were 0.74 (95% CI 0.70–0.78) and 0.68 (95% CI 0.62–0.74), for 15 studies using a cutoff threshold >12 mm were 0.73 (95% CI 0.68–0.77) and 0.69 (95% CI 0.61–0.75).

As considerable heterogeneity existed among included studies, we performed meta-regression to investigate the sources. Concerning studies using LCC, only publication year (≤2017 *vs.* >2017, P=0.02) was significantly associated with heterogeneity. Other factors such as length of LCC (≤10 *vs.* >10 mm and ≤12 *vs.* >12 mm), malignant rate (≤30 *vs.* >30%), study design (prospective *vs.* retrospective), magnet field strength (1.5 *vs.* 3.0 T), number of readers (<2 *vs.* ≥2), number of patients (≤150 *vs.* >150), and the publication year (≤2017 *vs.* >2017) were not significant factors contributing to heterogeneity, with ***P*** ranging from 0.11 to 0.96. For studies using other quantitative metrics, no significant factor was found substantially associated with heterogeneity, which are demonstrated in **Supplementary Table 2**.

## Discussion

In this meta-analysis, we investigated the diagnostic performance of several quantitative metrics with mpMRI for prediction of EPE at radical prostatectomy. The summary estimates of sensitivity and specificity for 16 studies using LCC were 0.79 (95% CI 0.75–0.83) and 0.77 (95% CI 0.73–0.80), for seven studies using ADC were 0.71 (95% CI 0.50–0.86) and 0.71 (95% CI 059–0.81), for five studies using tumor size were 0.62 (95% CI 0.57–0.67) and 0.75 (95% CI 0.67–0.82), and for three studies using tumor volume were 0.77 (95% CI 0.68–0.84) and 0.72 (95% CI 0.56–0.83), respectively. As considerable heterogeneity was observed between studies, we performed meta-regression to explore the sources. Among the several potential factors, we found that only publication year (≤2017 *vs.* >2017) was the significant factor responsible for heterogeneity (P=0.02). As several studies provided head-to-head comparison between LCC and ADC, as well as between LCC and tumor size, we performed direct comparison in available studies. According to our analyses, LCC was significantly inferior to ADC in specificity but was superior to tumor size in sensitivity; nevertheless, both comparisons were based on merely three studies and need more large-sample studies to validate in future.

LLC is defined as the length of prostate tumor in contact with the capsule, and the rationale behind which is that greater LCC on histopathology correlates with higher probability of EPE (49). A prior meta-analysis investigated the diagnostic accuracy of using LCC as independent predictor for detection of EPE, in which the pooled sensitivity and specificity were 0.79 and 0.67 (50). As for ADC, studies revealed that as tumor grade increases, a trend of increasing cellular density, with loss of the normal glandular structures and a decrease in the extracellular space, limiting water diffusivity and yielding lower ADC values (51, 52). ADC value has been shown to inversely correlate with pathological stage (42, 53), and a previous study demonstrated that when combining ADC value with other clinical information, the pooled sensitivity and specificity were 0.85 and 0.71 (54). The rationale of using tumor volume as predictor of EPE is based on findings that the diameter of the index lesion has a strong correlation with tumor volume at radical prostatectomy (42, 55). We performed indirect comparisons between these quantitative metrics, and the results demonstrated that the pooled sensitivity from tumor size was significantly lower than LCC (P=0.002) and tumor volume (P=0.013). Moreover, our analyses showed that the pooled specificity in tumor size was substantially lower than tumor size (P=0.04). However, these results were obtained from indirect comparisons thus should be interpreted with caution.

Considering that different cutoff thresholds were used with respect to LCC and tumor size, multiple subgroup analyses were performed to account for various outcomes. When restricted subgroup analysis to six studies using a cutoff value ≤10 mm, the pooled sensitivity and specificity were 0.78 and 0.67. In contrast, a cutoff value >10 mm yielded slightly lower sensitivity (0.74) and equivalent specificity (0.68). Likewise, a cutoff threshold ≤12 mm yielded an equivalent diagnostic performance as compared with >12 mm, with sensitivity of 0.78 *vs.* 0.73 and specificity of 0.67 *vs.* 0.69. As for the tumor size, subgroup analysis suggested that using a tumor size of 15 mm yield a moderate diagnostic performance, with sensitivity of 0.67 and specificity of 0.70. When compared with the subjective assessment that mainly depends on radiologists’ personal pattern and experience, the quantitative analysis offers several potential advantages of improving accuracy, interobserver agreement, and histopathology correlation. However, different measurement methods and tools, as well as MRI techniques and sequences, all may affect the final results, then lead to widely varied optimal cutoff values (18, 19). Regarding LCC, the reported optimal cutoff values ranged from 6 to 20 mm, with corresponding sensitivity of 0.60–0.89 and specificity of 0.44–0.88. Nevertheless, no significant difference between these cutoff thresholds. As for tumor size, although the PI-RADS recommends 15 mm for prediction of EPE, two studies demonstrated that a cutoff value of 16–18 mm yielded the best diagnostic performance (27, 37). With regard to ADC, despite that most studies included used the mean value as the assessment, two studies reported that results from ADC ratio or ADC entropy were superior to ADC mean value for distinguishing malignant from benign (33, 35). Using tumor volume as assessment for prediction of EPE was reported by merely three studies, which may be that it is often time-consuming and may require postprocessing on an independent workstation (33, 39, 43).

Our study has some limitations. First, most studies included were retrospective in study design, which resulted in a high risk regarding patient selection domain. Nevertheless, considering that nearly all studies available were retrospective, it was unfeasible to calculate summary estimates from the merely four prospective studies. Second, the heterogeneity was substantial among studies, which affected the general applicability of our study. We performed meta-regression and multiple subgroup analyses to explore the sources of heterogeneity; however, we found that most clinical covariates were not associated with the heterogeneity, thus a large proportion of which remains unexplained. Nonetheless, we applied a solid and robust methodology for this meta-analysis using the guidelines published by the Cochrane Collaboration. Third, the diagnostic results were extracted from the most accurate results; moreover, the size or length was measured using different MRI sequences or techniques. Last, the comparisons were based on indirect or merely several studies; thus, the results should be interpreted with caution.

## Conclusion

The mpMRI quantitative assessments of LCC, ADC, tumor size, and tumor volume showed moderate to high diagnostic performance in the prediction of EPE, of them LCC and tumor volume demonstrated higher accuracy than other assessments. However, the optimal cutoff threshold varied widely and should be established to apply them in clinical practice.

## Data Availability Statement

The original contributions presented in the study are included in the article/**Supplementary Material**. Further inquiries can be directed to the corresponding author.

## Author Contributions

Guarantor of the article: HTX. Conception and design: WL and YS. Collection and assembly of data: YMW and FL. Data analysis and interpretation: WL, YMW, and FL. All authors contributed to the article and approved the submitted version.

## Funding

This study was supported by the Natural Science Foundation of Jiangsu Vocational College of Medicine (No. 20204112).

## Conflict of Interest

The authors declare that the research was conducted in the absence of any commercial or financial relationships that could be construed as a potential conflict of interest.

## Publisher’s Note

All claims expressed in this article are solely those of the authors and do not necessarily represent those of their affiliated organizations, or those of the publisher, the editors and the reviewers. Any product that may be evaluated in this article, or claim that may be made by its manufacturer, is not guaranteed or endorsed by the publisher.
