# Supplementary material for: The Quantitative Assessment of Using Multiparametric MRI for Prediction of Extraprostatic Extension in Patients Undergoing Radical Prostatectomy: A Systematic Review and Meta-Analysis
Source: Front Oncol. 2021 Nov 22;11:771864. doi: 10.3389/fonc.2021.771864 (PMC8645791; doi:10.3389/fonc.2021.771864)
Supplement: Supplementary file 1 [file Table_1.docx]

Supplementary Table 1. Meta-regression

| **Covariates** | **Values** | **Sensitivity** | ***P*** | **Specificity** | ***P*** |
| --- | --- | --- | --- | --- | --- |
| ADC: Type | ADC mean | 0.71/0.40-0.88 | 0.96 | 0.70/0.53-0.81 | 0.77 |
|  | other | 0.72/0.54-0.94 |  | 0.74/0.65-0.89 |  |
| ADC: Patient Nol. | ＜150 | 0.65/0.27-0.86 | 0.46 | 0.77/0.57-0.87 | 0.27 |
|  | ＞150 | 0.79/0.72-0.94 |  | 0.65/0.58-0.79 |  |
| ADC: Country | Korea | 0.82/0.70-0.94 | 0.25 | 0.64/0.56-0.78 | 0.23 |
|  | Other | 0.61/0.28-0.83 |  | 0.77/0.57-0.87 |  |
| ADC: Year | ≤ 2016 | 0.82/0.72-0.93 | 0.14 | 0.65/0.59-0.78 | 0.21 |
|  | ＞2016 | 0.55/0.18-0.80 |  | 0.78/0.52-0.89 |  |
| Size | = 15 mm | 0.62/0.59-0.69 | 0.12 | 0.73/0.65-0.79 | 0.11 |
|  | ＞ 15 mm | 0.57/0.51-0.63 |  | 0.83/0.79-0.88 |  |
| LLC: Year | ≤ 2017 | 0.82/0.77-0.86 | 0.02 | 0.68/0.59-0.77 | 0.60 |
|  | ＞2017 | 0.73/0.68-0.78 |  | 0.65/0.56-0.73 |  |
| LLC: Patient No. | ＜150 | 0.75/0.68-0.80 | 0.32 | 0.65/0.53-0.81 | 0.64 |
|  | ＞150 | 0.79/0.75-0.83 |  | 0.68/0.62-0.76 |  |
| LLC: Mal rate | ＜30% | 0.78/0.72-0.84 | 0.56 | 0.68/0.63-0.77 | 0.58 |
|  | ≥ 30% | 0.76/0.71-0.81 |  | 0.65/0.53-0.73 |  |
| LLC: blind | Yes | 0.76/0.70-0.80 | 0.50 | 0.65/0.56-0.73 | 0.72 |
|  | Other | 0.78/0.72-0.83 |  | 0.68/0.58-0.76 |  |
| LLC: length | ≤ 10 mm | 0.78/0.69-0.84 | 0.64 | 0.67/0.56-0.77 | 0.94 |
|  | > 10 mm | 0.76/0.73-0.81 |  | 0.66/0.58-0.74 |  |
| LLC: length | ≤ 12 mm | 0.78/0.71-0.82 | 0.61 | 0.65/0.55-0.74 | 0.73 |
|  | > 12 mm | 0.76/0.72-0.81 |  | 0.68/0.60-0.76 |  |
| LLC: reader | < 2 | 0.79/0.73-0.86 | 0.68 | 0.73/0.53-0.84 | 0.35 |
|  | ≥ 2 | 0.77/0.72-0.81 |  | 0.65/0.58-0.72 |  |
| LLC: Design | Prospective | 0.78/0.73-0.84 | 0.75 | 0.64/0.56-0.75 | 0.58 |
|  | Retrospective | 0.76/0.71-0.81 |  | 0.68/0.59-0.74 |  |
| LLC: Magnet Field | 1.5 T | 0.80/0.73-0.86 | 0.42 | 0.65/0.49-0.77 | 0.79 |
|  | 3.0 T | 0.76/0.71-0.80 |  | 0.67/0.60-0.74 |  |

Supplementary Table 2. Indirect Comparison

| **Comparison** | ***P*** (sensitivity) | ***P*** (specificity) |
| --- | --- | --- |
| ADC vs. LLC | 0.47 | 0.42 |
| ADC vs. Size | 0.54 | 0.63 |
| ADC vs. Vol | 0.76 | 0.95 |
| LLC vs. Size | 0.002 | 0.18 |
| LLC vs. Vol | 0.67 | 0.45 |
| Size vs. Vol | 0.01 | 0.04 |
